# Pharmacokinetics and safety of gadopiclenol in Japanese healthy volunteers

**DOI:** 10.1007/s11604-025-01842-1

**Published:** 2025-07-29

**Authors:** Takashi Eto, Toshiaki Taoka, Mathieu Felices, Camille Pitrou

**Affiliations:** 1 Clinical Research Unit, Hakata Clinic, LTA Medical Co, Random Square 6-18, Tenyamachi, Hakata-Ku, Fukuoka, 812-0025 Japan; 2 Department of Innovative Biomedical Visualization, Nagoya University, 65 Tsurumai-Cho, Showa-Ku, Nagoya, Aichi 466-8550 Japan; 3 PhInC Development, 36, Rue Victor Basch, 91300 Massy, France; 4 Clinical Development Department, Guerbet, Roissy CDG Cedex, France

**Keywords:** Gadopiclenol, Macrocyclic, Magnetic resonance imaging, Pharmacokinetics, Safety

## Abstract

**Purpose:**

The aim of this study was to evaluate the pharmacokinetics and safety of gadopiclenol in Japanese healthy volunteers. A population-based pharmacokinetic approach was used to compare pharmacokinetic parameters with a non-Japanese adult population.

**Materials and methods:**

In this double-blind, placebo-controlled phase I study, Japanese healthy volunteers were randomized to receive gadopiclenol (at 0.025, 0.05, or 0.1 mmol/kg) or a placebo. Blood and urine samples were collected up to 24- and 48-h post-administration, respectively. The pharmacokinetic profile of gadopiclenol was evaluated using standard non-compartmental analysis. Adverse events (AEs) were collected during the whole study period.

**Results:**

Overall, 27 participants were randomized (median [range] age: 22 [20–43] years; 52% male): 18 received gadopiclenol (6 in each dose group), and 9 received the placebo. The mean systemic exposure of gadopiclenol increased proportionally with the injected dose (area under the curve [AUC]: 215–1034 μg/mL.h) and was comparable between the three dose groups when normalized to dose (AUC/dose: 182–189 μg/mL/g.h) and to non-Japanese (168–183 μg/mL.h). The mean terminal half-life (1.43–1.86 h), and the distribution volume (11.3–15.2 L) were also similar to those of non-Japanese healthy volunteers (1.50–1.73 h and 13.0–15.5 L, respectively). The mean fraction of gadopiclenol excreted in urine was between 87 and 95%, depending on the administered dose. Most of gadopiclenol (median of 95.7%) was excreted within 24 h after administration. The mean total clearance was comparable between the different administered doses (5.3–5.6 L/h) and similar to the mean renal clearance. No gadopiclenol-related AEs were reported.

**Conclusions:**

The pharmacokinetic profile of gadopiclenol is similar in Japanese and non-Japanese healthy volunteers. The population pharmacokinetic analysis showed no significant ethnic disparities between these two populations and suggested that no dose adjustment was required for Japanese patients. Gadopiclenol had a very good tolerability in Japanese healthy volunteers with no adverse reactions reported.

## Introduction

Contrast-enhanced magnetic resonance imaging (MRI) improves the visibility of specific tissues and organs, which is crucial to determine the extent of a disease and the effectiveness of a treatment in a variety of medical conditions [1–5].

Gadopiclenol (Elucirem™, Guerbet, France; Vueway, Bracco, USA) is a nonspecific macrocyclic gadolinium-based contrast agent (GBCA) characterized by its very high r1 relaxivity (i.e., 12.8/11.6 mM^−1^ s^−1^ in biological medium at 37 ℃, at 1.5/3 T) compared to other currently available GBCAs, such as gadobutrol (5.2/5 mM^−1^ s^−1^), gadoteridol (4.1/3.7 mM^−1^ s^−1^), or gadoterate (3.6/3.5 mM^−1^ s^−1^). In addition, gadopiclenol exhibits a very high kinetic stability under acidic conditions, with a dissociation half-life of 20 ± 3 days, and a lack of interaction with plasma proteins [6].

The safety profile of gadopiclenol has been investigated in a variety of non-Japanese populations, such as healthy volunteers [7–10], patients with various types of lesions [11, 12], including pediatric patients aged 2–17 years [13], and adult patients with various stages of renal impairment, including those with end-stage renal disease [8]. Depending on the studies, gadopiclenol has been tested at doses from 0.025 to 0.3 mmol/kg. All these studies highlighted a good safety profile of gadopiclenol, comparable to other macrocyclic GBCAs.

All currently available macrocyclic GBCAs are mainly excreted by kidneys, with an elimination half-life of approximately 1.7 h, and an extracellular distribution [14]. The pharmacokinetic profile of gadopiclenol was also investigated in several studies. In healthy volunteers, the pharmacokinetics of gadopiclenol are linear, with a terminal elimination half-life of 1.5 to 2 h, and has a relatively small distribution volume (i.e., 182–254 mL/kg), indicating that it is distributed within the extracellular compartment [7]. Gadopiclenol was shown to be mainly excreted in urine (98% of the administered dose recovered within 48 h after administration) in an unchanged form [7]. Nevertheless, the pharmacokinetic profile of gadopiclenol in the Japanese population had not yet been investigated.

Population pharmacokinetics modeling is a widely used method as a complement to usual pharmacokinetic methodologies for the evaluation of pharmacokinetics in clinical trials. It is used to calculate population parameters from sparse and imbalanced data from multiple studies, to assess the effect of age, disease state, or race on the pharmacokinetics of a specific drug, and eventually establish prescribing regimens for specific groups of patients [15].

Thus, the aim of this single ascending dose study was to evaluate the pharmacokinetic profile and safety of gadopiclenol following intravenous administration in Japanese healthy volunteers. A population pharmacokinetic analysis was also performed to assess any impact of ethnicity on the pharmacokinetics of gadopiclenol.

## Materials and methods

### Study design and population

This phase I, ascending dose, double-blind, randomized, placebo-controlled trial was conducted in a single center (Hakata Clinic, Fukuoka, Japan), between May and July 2021. The study was approved by an independent ethics committee, and written informed consent was obtained from each participant. The study was registered at https://jrct.niph.go.jp/ (registration n°: jRCT2071210029) and Clinicaltrials.gov (registration n°: NCT04906005).

Male or female Japanese healthy volunteers aged 20–60 years, with a body mass index (BMI) of 18–25 kg/m^2^ were included. A Japanese healthy volunteer was defined as being born in Japan and having both parents and four grandparents (maternal and paternal) who are ethnically Japanese and having Japanese lifestyle, including diet, as determined by participant’s verbal report. Good health status was determined by the investigator according to past medical history, clinical examination, including 12 lead ECG, vital signs (blood pressure, pulse rate, respiratory rate, and body temperature), and laboratory tests at screening and inclusion.

Participants were randomized and double-blindly administered with gadopiclenol (0.5 M solution) or placebo (0.9% sodium chloride solution). Three doses (0.025, 0.05, and 0.1 mmol/kg) were investigated. Nine participants were included in each dose group (27 participants in total): 6 received gadopiclenol and 3 received the placebo, as it was done in the phase I pharmacokinetics study conducted on non-Japanese healthy volunteers [7]. Dose escalation to the next dose group was only allowed if the clinical and biological safety of all participants from the previous dose group was acceptable.

Both products were administered as a single intravenous (IV) bolus injection at a rate of 2 mL/second followed by a saline flush using a power injector.

Participants were confined for one night before the inclusion visit and 2 days post-administration. A safety follow-up visit was performed 7 days after gadopiclenol or placebo administration.

### Pharmacokinetic assessments

The pharmacokinetic analysis was carried out with a non-compartmental method using Phoenix WinNonlin (Version 8.1; Pharsight Corporation, Mountain View, CA). The main calculated parameters were: area under the plasma concentration curve (AUC), plasma peak concentration (C_max_), terminal half-life (t_1/2_), volume of distribution (Vd), total clearance (CL), renal clearance (Clr), and gadopiclenol fraction excreted in urine (fe).

According to previous pharmacokinetic studies in non-Japanese (Caucasian) healthy volunteers, blood samples (6 mL) were drawn 30 min before, 2, 5, 10, 20, 30, and 45 min, and 1, 2, 4, 6, 8, 12, and 24 h after drug administration. Urine was collected 30 min before drug administration, and during the following time intervals: 0–6 h, 6–24 h, and 24–48 h after drug administration. For both blood and urine, an additional sample was collected during the follow-up safety visit. The determination of gadopiclenol concentration in plasma and urine was performed using a validated liquid chromatography with tandem mass spectrometric detection (LC–MS/MS) method, with a limit of quantification (LOQ) of 5 µg/mL. Gadopiclenol assay in plasma and urine and pharmacokinetic analysis were performed by Eurofins ADME-Bioanalyses (Vergèze, France).

### Safety evaluation

Vital signs (blood pressure, pulse rate, respiratory rate, and body temperature), electrocardiography (ECG), biochemistry, hematology, urinalysis, and estimated Glomerular Filtration Rate (eGFR) assessments were performed before and up to 48 h after drug administration, and during the safety follow-up visit. The eGFR was calculated using the Japanese coefficient-modified CKD-EPI formula [16]. Adverse events (AEs) were collected during the whole study period and tolerance at the injection site was monitored over 24 h after injection.

### Statistical methods

Descriptive summaries were performed using SAS (Version 9.4, SAS Institute Inc., Cary, NC, USA). Summary statistics (Number, Mean, Standard Deviation [SD], Median, Minimum, and Maximum) were presented for quantitative variables, and absolute and relative frequencies were presented for categorical variables.

A population pharmacokinetics modeling was performed to assess the impact of ethnicity on gadopiclenol pharmacokinetics. This was achieved using a Nonlinear Mixed Effect Modeling (NONMEM) software (v.7.4, Icon plc, Dublin, Ireland). The base model of the population pharmacokinetic analysis was obtained by combining data from Japanese participants in this study and others (non-Japanese adults with normal and impaired renal function, and pediatric patients from 2 to 17 years) who received gadopiclenol from three studies [7, 8, 13]. The population pharmacokinetic model that was previously developed in non-Japanese subjects was used to evaluate if it was predictive of Japanese subjects. Models evaluation was performed via NONMEM objective function and standard diagnostic or goodness of fit plots (predicted vs. observed, weighted residuals vs. predicted, weighted residuals vs. time). The first-order conditional estimation (FOCE) with interaction method was used. The covariate model was obtained using a univariate screening followed by a backward deletion process and should result in an improved fit. Validation of pharmacokinetic models was performed using visual predictive checks (VPC).

After preliminary checks and obtaining the final model, pharmacokinetic parameters (central volume of distribution [V1], peripheral volume of distribution [V2], total clearance [Cl], and terminal t_1/2_) were derived for Japanese and non-Japanese adults with normal renal function. In addition, gadopiclenol concentrations at 10, 20, and 30 min after administration and AUC for the doses 0.025 mmol/kg, 0.05 mmol/kg, and 0.1 mmol/kg were simulated in these two populations. One thousand replicates of each subject were obtained for each dose to determine the distribution of C10, C20, C30, and AUC. These exposures variables were compared by ethnic groups using descriptive statistics (minimum, 2.5th percentile, median, 97.5th percentile, geometric mean, and CV) as well as graphically using box-plots.

## Results

Overall, 104 healthy volunteers were screened, and 27 were randomized: 9 received the placebo, and 18 received gadopiclenol (6 in each dose group). All randomized participants completed the study. The reasons for screen failure (*n* = 52) consisted mainly in non-compliance with inclusion criteria (good health status, eGFR > 90 mL/min/1.73 m^2^), or consent’s withdrawal. Furthermore, 25 subjects included in the planned reserve did not need to be included in any dose group.

A major protocol deviation was reported for one participant who was randomized to the 0.025 mmol/kg dose group. For this participant, the actual volume of gadopiclenol administered was unknown. Therefore, this participant was excluded from the pharmacokinetic analysis which was performed on 26 participants.

A summary of the demographic characteristics of participants is provided in Table 1. Overall, participants were aged between 20 and 43 years with a median of 22 years, and the median weight was 58 kg. Participants were equally distributed according to sex.

**Table 1:** Demographic characteristics of participants

	Placebo (*N* = 9)	Gadopiclenol 0.025 mmol/kg (*N* = 6)	Gadopiclenol 0.05 mmol/kg (*N* = 6)	Gadopiclenol 0.1 mmol/kg (*N* = 6)	Total (*N* = 27)
*Age (years)*					
Mean (SD)	29.8 (9.4)	23.0 (4.6)	27.5 (6.1)	26.0 (8.2)	26.9 (7.6)
Median	30.0	21.5	28.5	21.5	22.0
Min; max	20; 43	20; 32	20; 36	20; 38	20; 43
*Sex*					
Male	5 (55.6%)	3 (50.0%)	3 (50.0%)	3 (50.0%)	14 (51.9%)
Female	4 (44.4%)	3 (50.0%)	3 (50.0%)	3 (50.0%)	13 (48.1%)
*Weight (kg)*					
Mean (SD)	57.66 (3.46)	54.23 (7.12)	58.63 (6.02)	59.53 (7.30)	57.53 (5.86)
Median	57.40	53.55	59.05	58.70	57.80
Min; max	51.1; 62.7	45.9; 62.4	50.3; 66.6	49.3; 72.0	45.9; 72.0
*Body Mass Index (kg/m*^*2*^*)*					
Mean (SD)	20.02 (1.27)	20.55 (1.93)	21.45 (1.91)	20.98 (1.51)	20.67 (1.63)
Median	20.50	20.35	21.85	20.70	20.50
Min; max	18.4; 22.0	18.8; 24.1	19.0; 23.8	19.4; 23.4	18.4; 24.1

*SD* Standard deviation

### Pharmacokinetics of gadopiclenol

Gadopiclenol was not quantifiable in either plasma or urine samples from participants who received the placebo.

**Fig. 1:**
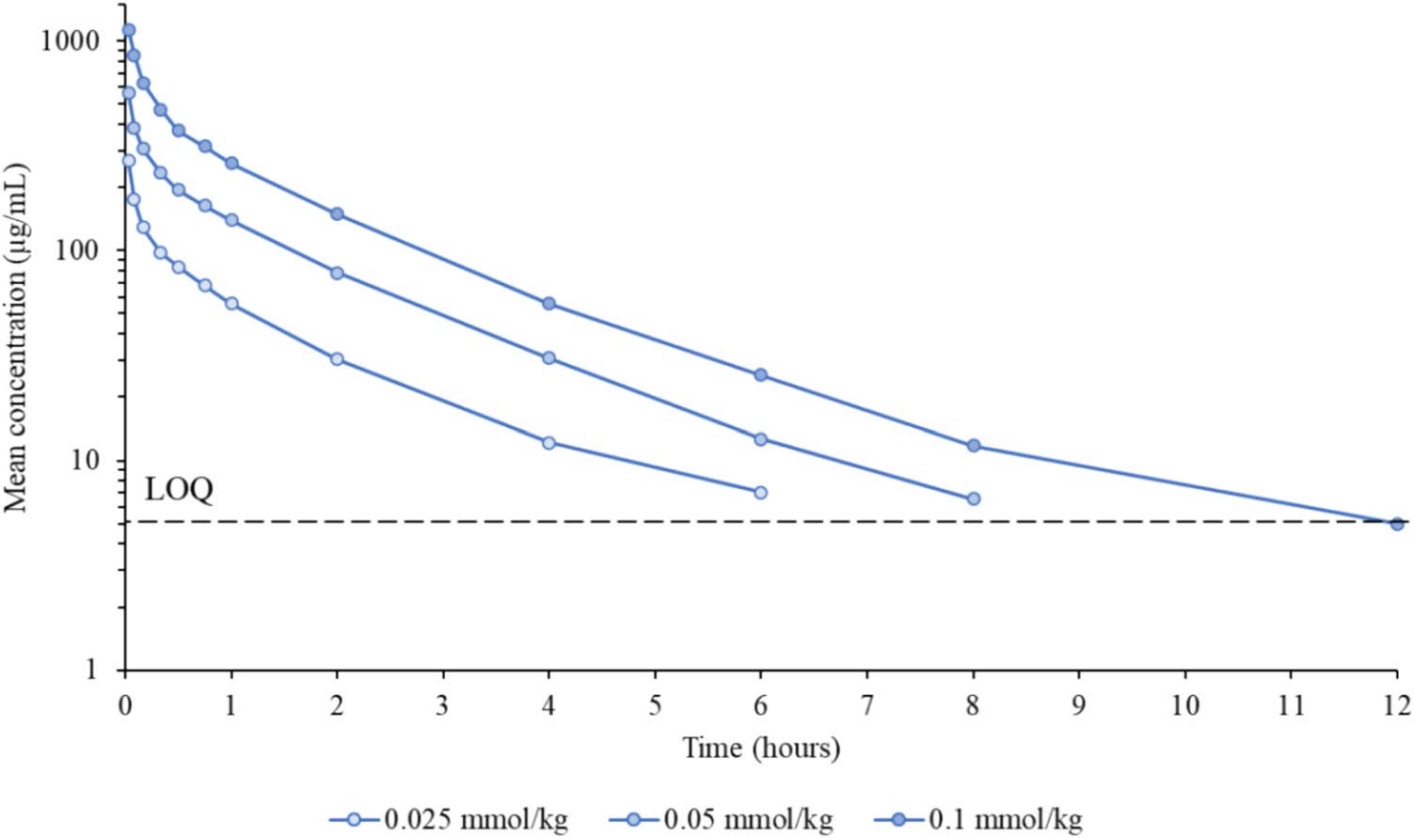
Time course of the mean plasma concentration of gadopiclenol in Japanese healthy participants. Mean gadopiclenol plasma concentration after single ascending intravenous doses (0.025, 0.05, and 0.1 mmol/kg) of gadopiclenol over time. LOQ: limit of quantification (5 µg/mL)

**Fig. 2:**
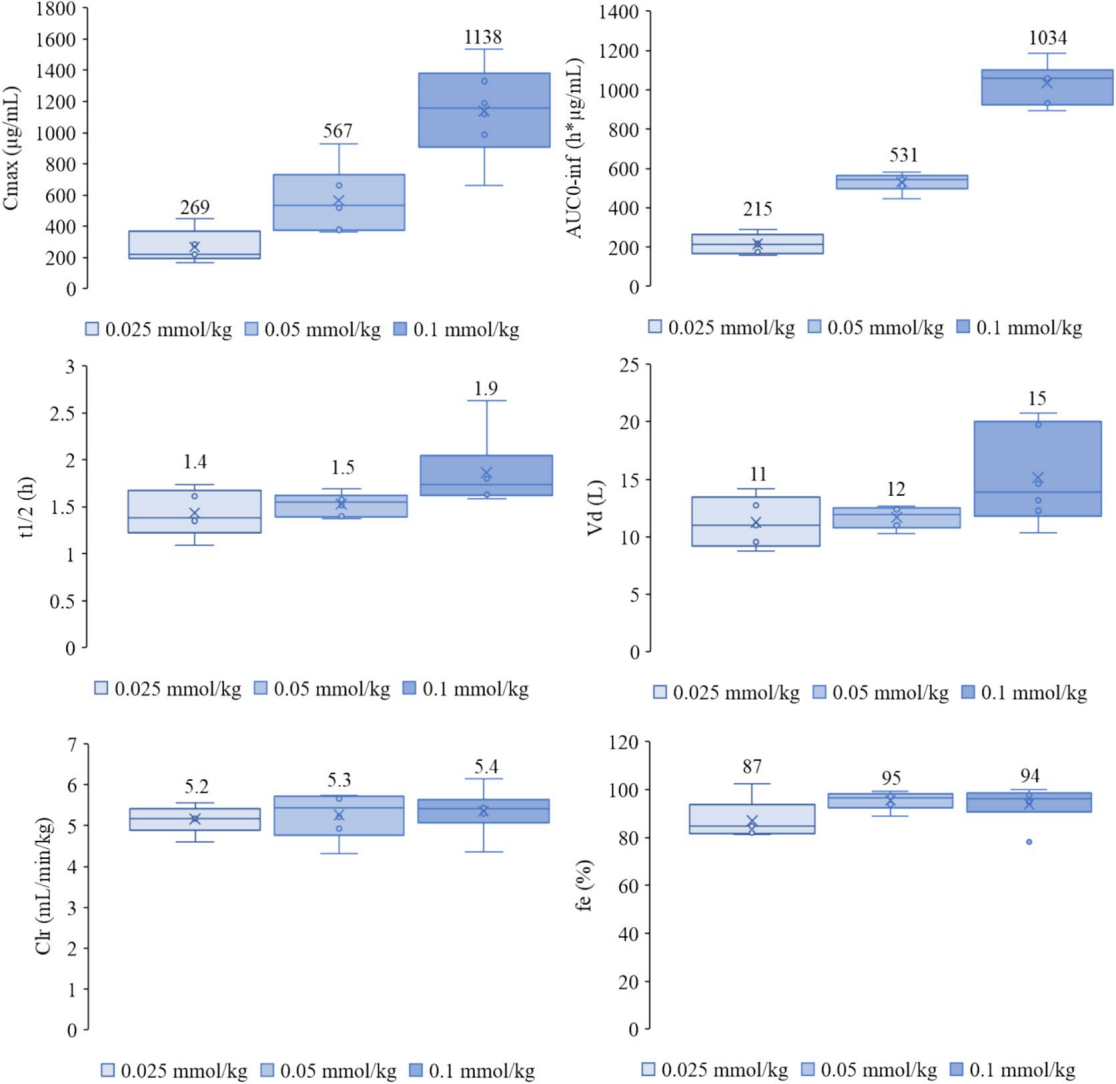
Pharmacokinetic parameters of gadopiclenol after intravenous administration in Japanese healthy participants. Box plot, representing the pharmacokinetics parameter of gadopiclenol administered as a single dose of 0.025, 0.05, or 0.1 mmol/kg: maximum plasma concentration measured (Cmax, µg/mL), area under the observed concentration–time curve from zero (time of drug administration) to infinite (AUC 0-inf, µg/mL.h, terminal elimination half-life of gadopiclenol (t1/2, h), volume of distribution (Vd, L), renal clearance (Clr, mL/min/kg), and percentage of gadopiclenol excreted in urine (fe, %). The arithmetic mean is represented by the cross and value, the median by the solid line, and the first and third quartile by the ends of the ‘Box’. The whiskers show the lowest data value still within 1.5 IQR (interquartile range). Data values that do not fall between the whiskers are plotted as outliers (markers outside of the whiskers). *C*_*max*_ peak concentration, *AUC*_*0*-inf_ area under the plasma concentration curve from injection to infinity, *t*_*1*/2_ terminal half-life, *Vd* distribution volume, *Clr* renal clearance

The time course of mean gadopiclenol plasma concentration after a single IV administration of gadopiclenol at 0.025, 0.5, and 0.1 mmol/kg in healthy Japanese participants is presented in Fig. 1. A summary of the main pharmacokinetic parameters measured in these participants is provided in Fig. 2. The mean AUC extrapolated to infinity increased proportionally with a AUC ratio close to 2 for each twofold increase of the injected dose and ranged between 215 (for 0.025 mmol/kg) and 1034 h × μg/mL (for 0.1 mmol/kg). The mean AUC extrapolated to infinity normalized to dose was comparable between 0.025, 0.05, and 0.1 mmol/kg doses (182 to 189 μg/mL/g.h), indicating that gadopiclenol pharmacokinetics are dose-independent.

The mean t_1/2_ was between 1.43 and 1.86 h and the Vd was between 11.3 and 15.2 L.

The median [Q1;Q3] t_1/2_ was between 1.44 [1.42; 1.46] and 1.7 [1.6; 1.8] h and the Vd was between 11.0 [9.6; 12.7] and 13.9 [12.3; 19.8] L.

The median fraction of gadopiclenol excreted in urine was between 84.6 [82; 84.7] and 96.5% [93.7; 97.8], depending on the administered dose.

Most of gadopiclenol was excreted within 24 h after administration (median of 95.7%, range: 77.6% to 100%). Gadopiclenol concentration in urine, measured 48 h after administration, was below LOQ for all samples except for two participants who received gadopiclenol at 0.1 mmol/kg for whom it was close to the LOQ (i.e., 5.5 and 6.9 µg/mL, respectively). The mean total clearance was comparable between the different administered doses (5.3–5.6 L/h) and similar to the mean renal clearance (5.2–5.4 L/h).

Considering the physiological coherence of the values obtained for the AUCinf/dose and for the clearance, the pharmacokinetic parameters appear similar to those obtained in the previous studies in non-Japanese subjects regarding the elimination of the drug (Table 2).

**Table 2:** Summary of gadopiclenol pharmacokinetic parameters across studies in non-Japanese and Japanese healthy volunteers—non-compartmental analyses

Gadopiclenol dose		0.025 mmol/kg		0.05 mmol/kg		0.1 mmol/kg		
Study		Non-Japanese [7]	Japanese	Non-Japanese [7]	Japanese	Non-Japanese [7]	Non-Japanese* [8]	Japanese
n subjects		5	5	6	6	6	8	6
Cmax (µg/mL)	Mean ± SD	248.7 ± 54.5	268.6 ± 109.5	524.5 ± 69.9	566.5 ± 209.5	992.0 ± 233.1	925.7 ± 291.1	1137.7 ± 298.2
	Median [Q1; Q3]	228.8 [209.4; 248.7]	222.5 [221.3; 285.3]	527.5 [456.2; 541.7]	532.9 [376.6; 662.6]	936.15 [786.2; 1250.5]	830.3 [803.1; 1050.8]	1155 [988.3; 1329.7]
AUCinf (µg/mL.h)	Mean ± SD	316 ± 28	215.2 ± 51.0	569 ± 102	530.58 ± 47.29	1288 ± 184	1112.6 ± 265.1	1034.2 ± 106.1
	Median [Q1; Q3]	315 [297; 332]	215.1 [175.8; 238.3]	588 [496; 605]	544.7 [511.1; 558.3]	1290 [1171; 1464]	1044.9 [944.6; 1056.7]	1060.6 [930.5; 1073.6]
AUCinf/Dose (µg/mL.h)	mean ± SD	183 ± 17	185.7 ± 24.5	168 ± 15	189.2 ± 22.2	176 ± 21	175.9 ± 19.6	182.4 ± 29.2
	Median [Q1; Q3]	183 [171; 194]	181.3 [164.4; 196.8]	172.5 [152; 178]	187.6 [175.6; 199]	179.5 [155; 190]	169.4 [163.2; 190.1]	183.6 [181.8; 191.9]
t1/2 (h)	mean ± SD	1.65 ± 0.43	1.43 ± 0.25	1.50 ± 0.22	1.53 ± 0.12	1.73 ± 0.26	1.92 ± 0.74	1.86 ± 0.39
	Median [Q1; Q3]	1.44 [1.42; 1.62]	1.38 [1.35; 1.61]	1.53 [1.28; 1.69]	1.5 [1.4; 1.6]	1.69 [1.5; 1.87]	1.77 [1.34; 2.22]	1.7 [1.6; 1.8]
Vd (L)	mean ± SD	13.2 ± 4.1	11.3 ± 2.2	13.0 ± 1.7	11.7 ± 1.0	14.4 ± 2.7	15.5 ± 4.4	15.2 ± 4.2
	Median [Q1; Q3]	12.1 [11.3; 12.63]	11.0 [9.6; 12.7]	13.2 [12.0; 14.2]	11.9 [11; 12.5]	14.0 [12;2; 16.0]	15.5 [11.7; 17.1]	13.9 [12.3; 19.8]
Cl (L/h)	mean ± SD	5.5 ± 0.5	5.5 ± 0.7	6.0 ± 0.6	5.3 ± 0.6	5.8 ± 0.7	5.7 ± 0.6	5.6 ± 1.0
	Median [Q1; Q3]	5.5 [5.2; 5.8]	5.5 [5.1; 6.1]	5.8 [5.6; 6.6]	5.3 [5; 5.7]	5.6 [5.3; 6.4]	5.9 [5.3; 6.1]	5.4 [5.2; 5.5]
Clr (L/h)	mean ± SD	5.3 ± 0.6	5.2 ± 0.3	5.2 ± 1.8	5.3 ± 0.6	6.0 ± 0.9	5.6 ± 1.0	5.4 ± 0.6
	Median [Q1; Q3]	5.5 [5.1; 5.7]	5.2 [5.2; 5.3]	5.3 [3.9; 6.7]	5.4 [4.9; 5.7]	5.9 [5.9; 6.7]	5.6 [4.8; 6.1]	5.4 [5.3; 5.5]
fe (%)*	mean ± SD	95.7 ± 7.6	87.0 ± 8.8	85.2 ± 25.7	95.4 ± 3.8	103.5 ± 11.8	97.3 ± 12.4	93.9 ± 8.0
	Median [Q1; Q3]	98.7 [88.7; 100.6]	84.6 [82; 84.7]	94 [69.2; 99.4]	96.5 [93.7; 97.8]	103.1 [101.3; 111.4]	97.8 [87.5; 104.6]	96.3 [94.6; 98.1]

Results are presented as mean ± SD and median [Q1-Q3]

*AUCinf* area under the plasma concentration curve from zero to infinity, *Cl* clearance, *Clr* renal clearance, *Cmax* plasma peak concentration, *t*_*1/2*_ terminal half-life, *Vd* volume of distribution, *fe* fraction excreted in urine

^*^Up to 48 h

Preliminary checks of the population pharmacokinetics model, previously developed for non-Japanese subjects, confirmed that it appeared suitable for the Japanese population if adjusted to take into account the differences between the populations. This model was a 2-compartment model with linear elimination from the central compartment in which all parameters were scaled to body weight using standard allometric rules with exponents 0.75 for clearances and 1 for volume. Only the eGFR was evidenced as significant covariate. Once pooled, an ethnic group covariate was maintained in the model to compare Japanese with non-Japanese subjects (no other covariate was included in the model). Estimated pharmacokinetic parameters from the final model showed slightly lower values for Cl and V1 in Japanese subjects compared to non-Japanese subjects, essentially due to body weight difference between the two populations (median of 58.8 kg for Japanese versus 72.6 kg for non-Japanese subjects). The median t_1/2_ was also slightly lower for Japanese subjects than for non-Japanese subjects, but this difference was pharmacokinetically irrelevant (Table 3). The parameters determined by the non-compartmental analysis and population approach (Vd, t_1/2_, Cl), were consistent, which confirmed the validity of the model. Overall, the median (9th percentile; 95th percentile) Cl and terminal half-life estimated from the final model were 5.6 (4.7–6.8) L/h and 1.5 (1.3–1.7) h for Japanese subjects and 6.3 (5.0–7.5) L/h and 1.8 (1.3–2.4) h for non-Japanese subjects, similar to the median values obtained in non-compartmental pharmacokinetic analyses (5.3–5.5 L/h and 1.4–1.7 h, respectively, for Japanese subjects and 5.5–5.9 L/h and 1.4–1.8 h, respectively, for non-Japanese subjects).

**Table 3:** Derived gadopiclenol pharmacokinetic parameters based on final population pharmacokinetic model

	Japanese	Non-Japanese*
Cl (L/h)	5.6 (4.7–6.8)	6.3 (5.0–7.5)
V1 (L)	5.0 (3.4–6.9)	7.6 (5.5–13.6)
V2 (L)	5.6 (4.6–7.3)	4.8 (3.2–8.0)
t_1/2_ (h)	1.5 (1.3–1.7)	1.8 (1.3–2.4)

Data presented as median (5th percentile – 95th percentile)

*Cl* clearance, *t*_*1/2*_ terminal half-life, *V1* central volume of distribution, *V2* peripheral volume of distribution

^*^Non-Japanese adults with normal renal function included in previous studies [7, 8]

**Fig. 3:**
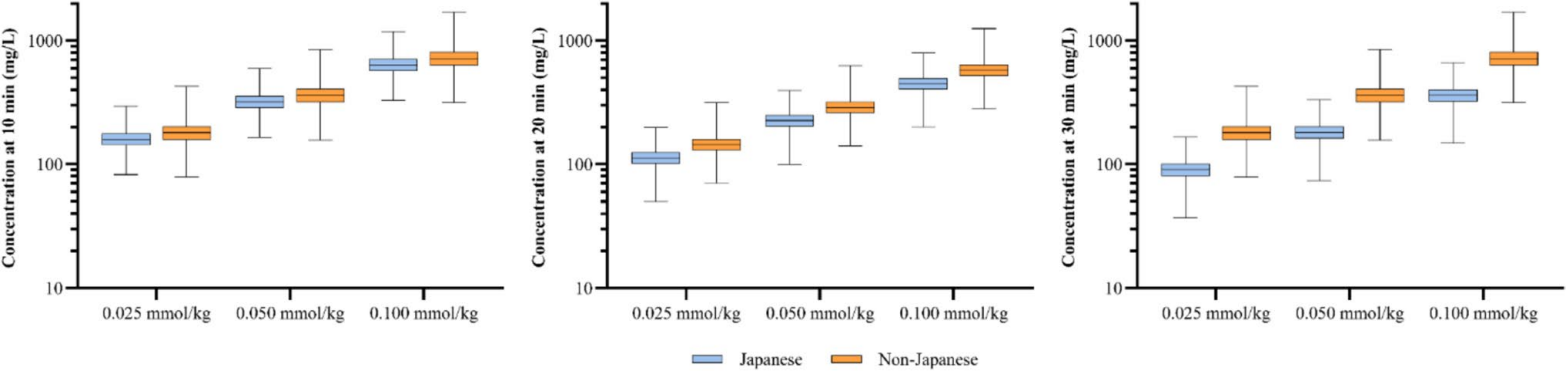
Simulated gadopiclenol plasma concentrations in Japanese and non-Japanese population. Box plot representation of simulated plasma concentration (mg/L) of gadopiclenol after a single intravenous injection at a dose of 0.025, 0.05, or 0.1 mmol/kg in Japanese and Non-Japanese populations with normal renal function. In the box plot, the median is represented by the solid line, and the first and third quartile by the ends of the “box”. The whiskers show the lowest value still within 1.5 interquartile range (IQR) of the lower quartile, and the highest value still within 1.5 IQR of the upper quartile

**Fig. 4:**
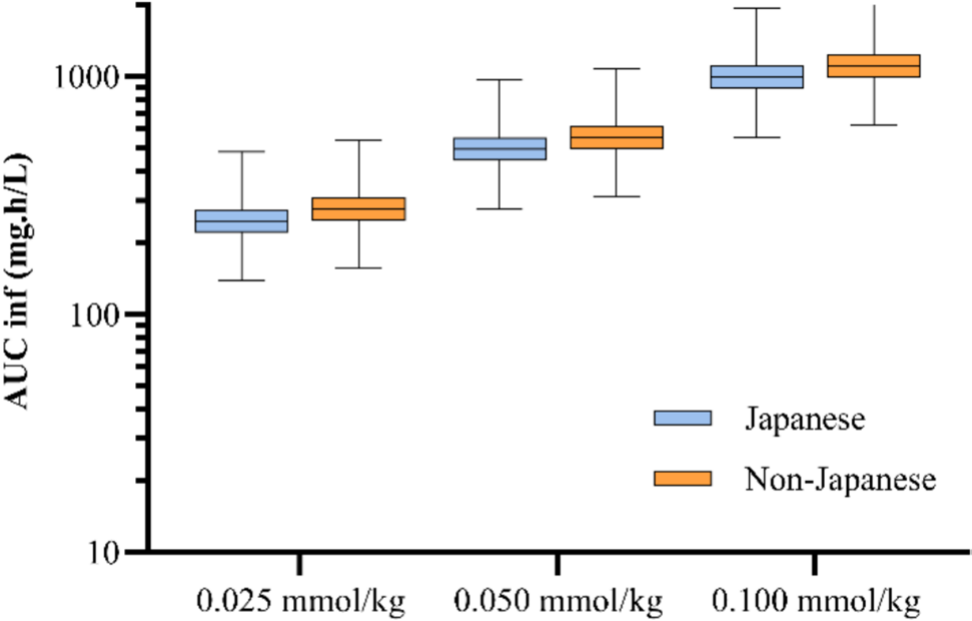
Simulated total systemic exposure of gadopiclenol in Japanese and non-Japanese population. Box plot representation of simulated AUC inf (mg/L.h) of gadopiclenol after a single intravenous injection at a dose of 0.025, 0.05, or 0.1 mmol/kg in Japanese and Non-Japanese populations with normal renal function. The median is represented by the solid line, and the first and third quartile by the ends of the “box”. The whiskers show the lowest value still within 1.5 interquartile range (IQR) of the lower quartile, and the highest value still within 1.5 IQR of the upper quartile

Similarly, the simulations performed based on the final population pharmacokinetic model showed that the median concentrations at 10, 20, and 30 min post-injection and AUC following a single administration of 0.025 mmol/kg, 0.05 mmol/kg, and 0.1 mmol/kg of gadopiclenol were slightly lower in Japanese than in non-Japanese adults with normal renal function (Figs. 3, 4). In all cases, these differences were limited and not pharmacokinetically significant (< 25%) and essentially due to differences between the two populations in body weight. To quantify the effect of ethnicity, additional simulations were performed on Japanese and non-Japanese populations presenting the same body weight and eGFR values. These simulations confirmed that ethnicity did not have a relevant pharmacokinetic impact on gadopiclenol exposure. The difference in median AUCinf between Japanese and non-Japanese subjects was reduced by 7% compared to previous simulations (difference of − 11%, whatever the dose). The median C10, C20, and C30 remained slightly lower for Japanese subjects than for non-Japanese subjects, by 10%, 21%, and 22%, respectively, whatever the dose.

### Safety evaluation

Two healthy participants reported AEs of mild intensity after placebo administration (22.2%): nausea and vomiting for one, and headache for the other. Among those who received gadopiclenol, only a medication error was reported in one participant (16.7%) randomized to the 0.025 mmol/kg dose group. For this participant, the investigator was not sure of the administered dose of gadopiclenol. No injection site reactions were reported. No clinically significant changes in laboratory parameters (i.e., blood chemistry, hematology, and urinalysis), vital signs, or cardiac rhythm were observed. No clinically significant decrease in eGFR was reported.

## Discussion

Gadopiclenol is a nonspecific, non-protein-binding, paramagnetic, macrocyclic, nonionic, high-relaxivity GBCA, approved by the US Food and Drug Administration in 2022 and European Commission in 2023. The efficacy of gadopiclenol in central nervous system (CNS) and body imaging has been demonstrated in two phase III studies [10, 11]. These two international, randomized, double-blinded, controlled, cross-over studies showed that contrast-enhanced MRI with gadopiclenol at half the gadolinium dose (i.e., 0.05 mmol/kg) was not inferior to that with gadobutrol at the standard dose of 0.1 mmol/kg, in terms of contrast enhancement, border delineation and visualization of the internal morphology of lesions located in the CNS [11] or other body regions, such as head and neck, thorax (including breast), abdomen (including liver), pelvis, and musculoskeletal [10].

The previous pharmacokinetic study in non-Japanese healthy volunteers showed that, like other extracellular GBCAs, after intravenous administration, gadopiclenol is distributed in the extracellular fluids, is not metabolized, and is mainly excreted in urine [7]. In the current study, the pharmacokinetics and safety of gadopiclenol, administered as a single bolus at the dose of 0.025, 0.05, and 0.1 mmol/kg, were specifically evaluated in Japanese healthy volunteers.

The Vd of gadopiclenol in Japanese healthy volunteers (ranging from 11.3 to 15.2 L depending on the dose) were comparable to that observed in non-Japanese healthy volunteers [7], and evocative of an extracellular distribution. The t_1/2_ (1.43–1.86 h) were also similar to those observed in non-Japanese healthy volunteers (1.50–1.73 h) [7]. The renal clearance of gadopiclenol was almost identical to the total clearance, indicating that, as for non-Japanese healthy volunteers [7], unchanged urinary excretion is the elimination route for gadopiclenol in Japanese healthy participants. Furthermore, the fraction of gadopiclenol excreted in urine within 48 h reported in Japanese healthy volunteers was close to complete (median of 85% to 97%) and comparable to that reported with non-Japanese healthy volunteers (median of 94% to 99%) [7].

Overall, these pharmacokinetic parameters were in line with those of other GBCAs such as gadobutrol or gadoterate meglumine [17, 18]. As it was performed on healthy volunteers, the current study included only participants with normal renal function (eGFR ≥ 90 mL/min). Nevertheless, the pharmacokinetics of gadopiclenol were previously investigated in non-Japanese patients with mild (eGFR: 60–89 mL/min), moderate (eGFR: 30–59 mL/min), or severe (eGFR: 15–29 mL/min) renal impairment [8]. Gadopiclenol t_1/2_ was prolonged in these patients, yet the renal clearance of gadopiclenol was complete or nearly complete [8].

In addition to the non-compartmental approach, the pharmacokinetic parameters of gadopiclenol in Japanese healthy volunteers were also investigated using a population pharmacokinetic approach. This approach was used to assess the need for dose adjustment in the Japanese population. Similarly to other macrocyclic GBCAs [19, 20], the current study showed that gadopiclenol pharmacokinetics in Japanese healthy volunteers can be adequately described with a 2-compartment model with linear elimination from the central compartment. The simulated gadopiclenol concentrations based on this model showed limited differences, that were essentially attributed to differences in body weight and estimated intercompartment clearance between Japanese and non-Japanese participants rather than inherent ethnic factors. This was confirmed with further simulations taking into account only participants with equivalent body weight and eGFR values. Overall, these data indicate that dose adaptation of gadopiclenol is not necessary for the Japanese population. To our knowledge, there are no published studies comparing the pharmacokinetics of GBCAs between Japanese and non-Japanese subjects. However, it is worth mentioning that the approved dosage of the macrocyclic GBCAs commercialized in Japan is not different from that in US or Europe.

Based on our findings showing the similar pharmacokinetics of gadopiclenol between Japanese and non-Japanese populations, the efficacy of gadopiclenol (i.e., contrast enhancement) should theoretically be comparable between these two populations.

The safety profile of gadopiclenol had been evaluated in 8 prior clinical studies with 1047 participants exposed to gadopiclenol [21]. These studies included healthy volunteers, adult or pediatric patients aged 2–17 years undergoing MRI of the CNS or other body regions, as well as adult patients with impaired renal function. All these studies highlighted the good safety profile of gadopiclenol, with an overall incidence of AEs comparable to that of other GBCAs. The most frequent AEs related to gadopiclenol were various types of injection site reactions, headache, and nausea [21]. Nephrogenic systemic fibrosis is a rare fibrosing disorder primarily affecting the skin but also other organs, such as the liver, heart, muscles, and lungs, which has been associated with GBCAs’ administration [22]. So far, no case of nephrogenic systemic fibrosis has been reported in patients who have received gadopiclenol. This is in line with the previous reports showing that most NSF cases were observed after the use of linear GBCAs, while only few unconfounded cases were reported with macrocyclic GBCAs [23]. The current study also highlighted the good tolerability of gadopiclenol, with no adverse reactions reported and no clinically significant changes in laboratory parameters or cardiac rhythm.

Limitations of that study are those inherent to phase I studies: a limited sample size and the inclusion of healthy volunteers with normal renal function. Specific populations such as patient population, pediatric and above 60 of age or patients with BMI above 25 kg/m^2^ are not represented in this study. Only descriptive statistics were used and no statistical comparison was planned. However, the design of the study, placebo-controlled, allowed to provide pharmacokinetic assessments to evaluate the appropriate dose for further clinical use in Japanese population. The population pharmacokinetic model included participants from different studies, and the population outside Japan included in the model was more heterogeneous than the Japanese population from the phase I study. However, all had similar collection and analysis of samples.

In conclusion, the pharmacokinetics of gadopiclenol in Japanese healthy volunteers are dose-independent in the dose range of 0.025–0.1 mmol/kg. The population pharmacokinetic analysis indicated that there are no significant ethnic disparities in the pharmacokinetics of gadopiclenol between Japanese and non-Japanese populations, with only small non-clinically relevant differences, suggesting that no dose adjustment is required. The present study also demonstrated the good tolerance of gadopiclenol in the Japanese population.

## References

[1] Wattjes MP, Ciccarelli O, Reich DS, Banwell B, de Stefano N, Enzinger C, et al. 2021 MAGNIMS-CMSC-NAIMS consensus recommendations on the use of MRI in patients with multiple sclerosis. Lancet Neurol. 2021;20(8):653–70. 34139157 10.1016/S1474-4422(21)00095-8

[2] Hemond CC, Bakshi R. Magnetic resonance imaging in multiple sclerosis. Cold Spring Harb Perspect Med. 2018. 10.1101/cshperspect.a028969. 29358319 10.1101/cshperspect.a028969 PMC5932576

[3] Bonm AV, Ritterbusch R, Throckmorton P, Graber JJ. Clinical imaging for diagnostic challenges in the management of gliomas: a review. J Neuroimaging. 2020;30(2):139–45. 31925884 10.1111/jon.12687 PMC8300867

[4] Mitchell DK, Kwon HJ, Kubica PA, Huff WX, O’Regan R, Dey M. Brain metastases: an update on the multi-disciplinary approach of clinical management. Neurochirurgie. 2022;68(1):69–85. 33864773 10.1016/j.neuchi.2021.04.001 PMC8514593

[5] Niendorf E, Spilseth B, Wang X, Taylor A. Contrast enhanced MRI in the diagnosis of HCC. Diagnostics. 2015;5(3):383–98. 26854161 10.3390/diagnostics5030383 PMC4665604

[6] Robic C, Port M, Rousseaux O, Louguet S, Fretellier N, Catoen S, et al. Physicochemical and pharmacokinetic profiles of Gadopiclenol: a new macrocyclic gadolinium chelate with high T1 relaxivity. Invest Radiol. 2019;54(8):475–84. 30973459 10.1097/RLI.0000000000000563 PMC6661244

[7] Hao J, Bourrinet P, Desche P. Assessment of pharmacokinetic, pharmacodynamic profile, and tolerance of Gadopiclenol, a new high relaxivity GBCA, in healthy subjects and patients with brain lesions (Phase I/IIa study). Invest Radiol. 2019;54(7):396–402. 30870257 10.1097/RLI.0000000000000556

[8] Bradu A, Penescu M, Pitrou C, Hao J, Bourrinet P. Pharmacokinetics, dialysability, and safety of Gadopiclenol, a new gadolinium-based contrast agent, in patients with impaired renal function. Invest Radiol. 2021;56(8):486–93. 34197356 10.1097/RLI.0000000000000764

[9] Funck-Brentano C, Felices M, Le Fur N, Dubourdieu C, Desche P, Vanhoutte F, et al. Randomized study of the effect of gadopiclenol, a new gadolinium-based contrast agent, on the QTc interval in healthy subjects. Br J Clin Pharmacol. 2020;86(11):2174–81. 32302009 10.1111/bcp.14309 PMC7576613

[10] Kuhl C, Csoszi T, Piskorski W, Miszalski T, Lee JM, Otto PM. Efficacy and safety of half-dose gadopiclenol versus full-dose gadobutrol for contrast-enhanced body MRI. Radiology. 2023;308(1): e222612. 37462494 10.1148/radiol.222612

[11] Loevner LA, Kolumban B, Hutoczki G, Dziadziuszko K, Bereczki D, Bago A, et al. Efficacy and safety of Gadopiclenol for contrast-enhanced MRI of the central nervous system: the PICTURE randomized clinical trial. Invest Radiol. 2023;58(5):307–13. 36729404 10.1097/RLI.0000000000000944 PMC10090311

[12] Bendszus M, Roberts D, Kolumban B, Meza JA, Bereczki D, San-Juan D, et al. Dose finding study of Gadopiclenol, a new macrocyclic contrast agent, in MRI of central nervous system. Invest Radiol. 2020;55(3):129–37. 31917762 10.1097/RLI.0000000000000624

[13] Jurkiewicz E, Tsvetkova S, Grinberg A, Pasquiers B. Pharmacokinetics, safety, and efficacy of Gadopiclenol in pediatric patients aged 2 to 17 years. Invest Radiol. 2022;57(8):510–6. 35318970 10.1097/RLI.0000000000000865 PMC9390233

[14] Layne KA, Dargan PI, Archer JRH, Wood DM. Gadolinium deposition and the potential for toxicological sequelae - A literature review of issues surrounding gadolinium-based contrast agents. Br J Clin Pharmacol. 2018;84(11):2522–34. 30032482 10.1111/bcp.13718 PMC6177715

[15] US Department of Health and Human Services FDA, Center for Drug Evaluation and Research (CDER), and Center for Biologics Evaluation and Research (CBER). Guidance for industry: population pharmacokinetics Accessed February 8, 2023 [Available from: https://www.fda.gov/media/128793/download.

[16] Horio M, Imai E, Yasuda Y, Watanabe T, Matsuo S. Modification of the CKD epidemiology collaboration (CKD-EPI) equation for Japanese: accuracy and use for population estimates. Am J Kidney Dis. 2010;56(1):32–8. 20416999 10.1053/j.ajkd.2010.02.344

[17] Staks T, Schuhmann-Giampieri G, Frenzel T, Weinmann HJ, Lange L, Platzek J. Pharmacokinetics, dose proportionality, and tolerability of gadobutrol after single intravenous injection in healthy volunteers. Invest Radiol. 1994;29(7):709–15. 7960618 10.1097/00004424-199407000-00008

[18] Le Mignon MM, Chambon C, Warrington S, Davies R, Bonnemain B. Gd-DOTA. Pharmacokinetics and tolerability after intravenous injection into healthy volunteers. Invest Radiol. 1990;25(8):933–7. 2394577

[19] Scala M, Koob M, de Buttet S, Bourrinet P, Felices M, Jurkiewicz E. A pharmacokinetics, efficacy, and safety study of Gadoterate Meglumine in pediatric subjects aged younger than 2 years. Invest Radiol. 2018;53(2):70–9. 28906338 10.1097/RLI.0000000000000412 PMC5768226

[20] Hahn G, Sorge I, Gruhn B, Glutig K, Hirsch W, Bhargava R, et al. Pharmacokinetics and safety of gadobutrol-enhanced magnetic resonance imaging in pediatric patients. Invest Radiol. 2009;44(12):776–83. 19858730 10.1097/RLI.0b013e3181bfe2d2

[21] Hao J, Pitrou C, Bourrinet P. A comprehensive overview of the efficacy and safety of Gadopiclenol: a new contrast agent for MRI of the CNS and body. Invest Radiol. 2024;59(2):124–30. 37812485 10.1097/RLI.0000000000001025 PMC11441729

[22] Mathur M, Jones JR, Weinreb JC. Gadolinium deposition and nephrogenic systemic fibrosis: a radiologist’s primer. Radiographics. 2020;40(1):153–62. 31809230 10.1148/rg.2020190110

[23] Food and Drug Administration. Gadolinium-based contrast agents (GBCAs) and the NSF risk: regulatory update. Accessed March 7, 2023.

